# On the Preparation of Deodorized Tincture of Opium

**Published:** 1867-06

**Authors:** Albert E. Ebert


### *On the Preparation of Deodorized Tincture of Opium.* By Albert E. Ebert.

Among the many new preparations incorporated in our present codex, and exemplifying the progress of pharmaceutical science, none, perhaps, was greeted with more satisfaction by physician and apothecary than the deodorized tincture of opium. Combining, in a liquid form, all of the narcotic properties of the drug, without the noxious, odorous and resinous principles, it is capable of producing the soporific effects of opium without subsequent prostration of the nervous system.

Notwithstanding the acknowledged advantages of the deodorized tincture, it has not been so generally used as was expected, and this fact may be partially explained, at least, by considerations of cost. Its expensiveness is due, in great measure, to the waste of ether employed in the process of the deodorization, for, though the separated portion of this liquid may be rendered available for subsequent use by distillation, this method of purification is not practicable with the majority of the pharmaciens, and without especial precaution it is attended with a risk greater than the value of the ether. These facts discourage the apothecary, and tend to place the preparation of the tincture solely in the hands of the wholesale manufacturer. With the view of regaining this ether without resort to distillation I made numerous experiments, and finally discovered a method by which the object may be accomplished at a trifling cost, and with little trouble. Upon the addition of a caustic alkali to the etherial solution the odorous, resinous, and coloring matters are nearly all withdrawn, and the ether is fitted for future use as a deodorizer. The process is as follows: Take of common caustic potassa one troyounce; place it in one pint of the ethereal solution, and agitate occasionally for twenty-four hours. Decant the ether, mix with four fluidounces of distilled water, allow it to separate; again remove the ether and keep it for further use.

While engaged in the foregoing manipulations, I conceived the idea of substituting for the ether the light product, obtained in the rectification of petroleum, known as "benzine." A trial of this solvent convinced me of its applicability, and after a series of carefully conducted experiments, I became convinced of its decided superiority. The results of my investigation, with the attending advantages, may be stated as follows:—The odorous and resinous matters in the aqueous solution of opium are more completely removed by benzine, while the morphia is not dissolved to a greater extent than by the use of ether.

Benzine does not extract codeia or narcotina; ether removes the former partially, and the latter altogether, from the solution. A practical advantage in the use of benzine is the facility with which it may be separated from the deodorized solution. It is only necessary to pour the mixture on a moistened paper filter, when the watery extract will rapidly pass, admixed with but a trace of benzine, which may be expelled by a comparatively slight application of heat. Economically considered, (its cost being but 1-25 that of ether) the advantage of using benzine is important, as its use may exert an influence on the general employment of deodorized tincture of opium. The following formula, involving the use of benzine, is nearly in the language of the Pharmacopœia.

### *Deodorized Tincture of Opium.*

Take of opium, dried, in moderately fine powder, 2½ troyounces; Benzine, sp. gr. ·700 to ·730, (or of such purity that, when dropped on white paper, and allowed to evaporate spontaneously, leave no stain;) Alcohol, each half pint; water a sufficient quantity. Macerate the opium with half a pint of water for twenty-four hours, and express; then repeat the operation twice with the same quantity of water; mix the expressed liquids, and, having evaporated the mixture to four fluidounces, shake it, when cold, in a bottle, repeatedly, with the benzine; allow it to stand for about eight hours, and separate; then pour the mixture on a paper filter, previously moistened with water. When all the watery solution has passed, decant the benzine, and wash the filter with a small quantity of water, so as to avoid loss; evaporate the liquid by a gentle heat, until all traces of benzine have disappeared; mix this with twenty fluidounces of water, allow the mixture to stand a few hours, and filter through paper; when the liquid has ceased to pass, add sufficient water through the filter to make the filtered liquid measure a pint and a half; lastly, add the alcohol, and mix them together.

It has long been admitted that aqueous solutions of opium act more favorably on the system than those prepared with alcohol, or than the drug itself. It has also been observed that the narcotic power of the aqueous preparations is not in exact proportion to the drug represented, being somewhat less. How far this diminished sedative effect, and the increased pleasantness of action, is due to the absense of the usual quantity of narcotina, (which is but imperfectly abstracted from opium water) or to the removal of the odorous, resinous and fatty principles, has not, so far as I am aware, been satisfactorily determined.

Deodorized tincture of opium, as before intimated, when made with ether, can contain but little narcotina, and only a portion of the codeia of the drug.

A literal interpretation of the officinal name would indicate a faulty nomenclature; for, beside being deprived of odor, at least one important alkaloid is, to a considerable extent, absent from the tincture.

A deodorized preparation, more nearly representing the alkaloids of opium, and consequently, better deserving the officinal *title*, but which, for distinction, I will designate *Purified Tincture of Opium*, may be made as follows:—

The first step of the process being the preparation of *deodorized opium*, which might prove a substitute for the expensive article known as "denarcotized opium."

*Deodorized Opium.*

Take of opium, in moderately fine powder, 2½ troyouuces; Benzine, sp. gr. ·700 to ·730, a pint; macerate the opium with half a pint of benzine for twelve hours; separate the benzine by decantation, and repeat the operation again with the same quantity of benzine; then pour it on a paper filter, and when the liquid has ceased to pass, dry it by means of a gentle heat.

*Purified Tincture of Opium.*

Take of deodorized opium, the product from 2½ troyounces of powdered opium; alcohol, water, each a pint; proceed as directed for preparing tincture of opium, U. S. P.

I suggest these new preparations with some hesitancy, and would not be understood as recommending untried substitutes for articles of ascertained value.

It is with the desire to further elucidate the therapeutics of this valuable drug that I advance these ideas, hoping, at some future time, to be able to add some experimental results to these theoretical speculations.—*American Journal of Pharmacy.*

